# Integrated Phenotypic and Whole-Genome Analysis of *Enterococcus hirae* HI3 Isolated from Hu Sheep Jejunum Supports Its Potential as a Ruminant Probiotic Candidate

**DOI:** 10.3390/microorganisms14081730

**Published:** 2026-08-06

**Authors:** Xin Song, Ying Guo, Foyang Zhou, Mengzhi Wang, Yujia Jing

**Affiliations:** College of Animal Science and Technology, Yangzhou University, Yangzhou 225009, China; songx0901@163.com (X.S.); 13511753776@163.com (Y.G.); 18936869896@163.com (F.Z.); mengzhiwang@yzu.edu.cn (M.W.)

**Keywords:** *Enterococcus hirae*, probiotic, whole-genome analysis, ruminant, acid and bile salt tolerance, bacteriocin, antibiotic susceptibility

## Abstract

The ban on antibiotic growth promoters in livestock production has created an urgent demand for safe and effective probiotic alternatives derived from indigenous animal microbiota. Here, we isolated *Enterococcus hirae* HI3 from Hu sheep jejunum and confirmed its species assignment via whole-genome sequencing, with ANI analysis showing 98.92% identity to the *E. hirae* reference genome. HI3 showed moderate acid tolerance, with survival rates of 19.31% (95% CI: 15.83–22.78%) at pH 2 and 18.40% (95% CI: 17.87–18.94%) at pH 3 after 4 h, and maintained > 50% survival in 2% bile salts. It was susceptible to penicillin, ampicillin, erythromycin, and chloramphenicol. No hemolytic activity was observed, and major enterococcal virulence genes (*gelE*, *cyl*, *esp*, *hyl*) were absent from the HI3 genome. The HI3 genome (3.06 Mb) consists of one circular chromosome and three circular plasmids. Functional annotation identified 229 carbohydrate metabolism genes (including 65 glycoside hydrolases) and 43 probiotic-related genes involved in acid tolerance (*atpA–G*, *nhaC*), bile salt tolerance (*cbh*), stress responses (*clp* family, *groEL*, *dnaK*, *cspA*, *sod2*), and adhesion (*ltaS*, *srtA*, *eno*, *epsA*). Multiple bacteriocin biosynthetic gene clusters (enterolysin A, class II lanthipeptide, etc.) were identified. Plasmid-borne tetracycline resistance genes *tet(M)* and *tet(L)* were detected; however, their functional transferability requires experimental validation. No other known transferable resistance determinants were identified in the genomic analysis. These *in vitro* and genomic findings represent an initial characterization of *E. hirae* HI3 and support its potential as a ruminant probiotic candidate. However, *in vivo* studies are required to validate its colonization capacity, safety, and efficacy in the target ruminant species.

## 1. Introduction

The widespread use of antibiotic growth promoters in animal husbandry has accelerated the emergence and dissemination of antimicrobial resistance, leading to global restrictions or outright bans on their application. Consequently, there is an urgent need for effective and safe alternatives that can maintain animal health and productivity without compromising food safety or public health. Ruminants possess a unique gastrointestinal ecosystem with physicochemical conditions (e.g., rumen pH, retention time, and fermentation profiles) distinct from monogastrics. Probiotics derived from the indigenous ruminant microbiota are better adapted to colonize and persist in this environment than strains of non-ruminant origin, supporting the rationale for host-matched probiotic development.

The intestinal microbiota plays an essential role in maintaining host health, including functions such as food digestion, vitamin synthesis, immune regulation, and pathogen defense [1,2]. Within this context, probiotics have emerged as promising live microbial agents capable of conferring beneficial effects on the host [3]. Their mechanisms of action include competitive exclusion of pathogens via nutrient and adhesion site competition [4]; modulation of host innate and adaptive immune responses [5]; and production of beneficial metabolites, such as short-chain fatty acids (SCFAs), which serve as energy substrates for intestinal epithelial cells and contribute to gut homeostasis [6]. These properties enable probiotics to play a critical role in preventing diarrhea, alleviating inflammation, and maintaining overall health.

The genus *Enterococcus* is a well-studied source of probiotic candidates. It comprises Gram-positive, facultatively anaerobic bacteria widely distributed in the gastrointestinal tracts of livestock, where they constitute a core commensal microbiota in the intestine [7]. Certain strains within this genus have been extensively validated as potential probiotic resources due to their advantageous characteristics, including antimicrobial activity, acid and bile salt tolerance, lysozyme resistance, and antibiotic susceptibility profiles [8,9,10]. Within this genus, *Enterococcus hirae* has emerged as a notable candidate. Since its first isolation from swine intestines and chicken crops [11], *E. hirae* has been continuously explored for its probiotic properties across various animal hosts, including ruminants, swine, poultry, and aquatic species, establishing it as a key target in probiotic screening.

Previous studies have established several probiotic-relevant traits of *E. hirae*. The species maintains high viability under simulated gastrointestinal conditions, with choloylglycine hydrolase and Na^+^/H^+^ antiporter systems constituting the core molecular basis for acid and bile salt tolerance [12,13]. Furthermore, certain *E. hirae* strains can secrete the bacteriocin nisin, which exhibits broad-spectrum stability and potent inhibitory activity against various pathogenic strains [14]. Recent research has also revealed that *E. hirae* can produce novel enterocins, such as enterocin E20c and enterocin LD3, with significant inhibitory effects against *Salmonella* [15,16]. Additionally, some strains produce exopolysaccharides (EPS) with reported antimicrobial, antioxidant, and prebiotic activities [17,18].

Despite these advances, research on ruminant-derived *E. hirae* remains limited. Most existing studies have focused on conventional physiological and biochemical characterization, and a systematic understanding of the genetic determinants underlying adhesion, stress tolerance, and antimicrobial biosynthesis in this species is lacking. Whole-genome sequencing, combined with comprehensive functional annotation, provides a powerful approach to elucidating the genetic basis of probiotic traits and assessing strain safety at the genomic level. Integrating phenotypic characterization with genomic analysis is therefore essential for the rigorous evaluation of *E. hirae* as a probiotic candidate.

In this study, an *E. hirae* strain, designated HI3, was isolated from the jejunum of Hu sheep. We systematically evaluated its growth characteristics, acid and bile salt tolerance, antibiotic susceptibility, and hemolytic activity. Whole-genome sequencing and functional annotation were performed to characterize its genomic architecture, identify genes associated with probiotic functions, and assess biosafety at the genomic level. This integrated approach aims to provide a scientific basis for the potential development of HI3 as a ruminant-specific probiotic candidate.

## 2. Materials and Methods

### 2.1. Isolation and Identification of Bacterial Strains

Jejunal chyme samples were collected from four healthy male Hu sheep (6 months old, weighing 45 ± 5 kg). All animals were clinically examined before sampling and showed normal body temperature, feed intake, and fecal consistency, with no signs of disease or medication use within the preceding 7 days. The animals were fed a total mixed ration with a concentrate-to-roughage ratio of 1:1, and were fasted for 12 h prior to euthanasia. Following the method of Maryam et al. [19], jejunal chyme samples were collected. The experiments were conducted according to the ethical policies and procedures approved by the Animal Care and Use Committee of Yangzhou University, Jiangsu, China (Approval No. 202503-234). The samples were serially diluted with sterile phosphate-buffered saline (PBS), and spread onto De Man, Rogosa, and Sharpe (MRS) agar plates. The plates were incubated at 37 °C for 24–48 h until colonies formed. Following incubation, ten colonies with typical enterococcal morphological characteristics were picked and repeatedly streaked onto fresh MRS agar plates to obtain pure cultures. These isolates were then subjected to Gram staining and molecular identification based on 16S rRNA gene sequencing. Among them, one isolate, identified as Gram-positive and confirmed as *Enterococcus hirae*, was selected for further characterization based on its preliminary growth performance and designated as strain HI3. The purified strain was preserved at −80 °C in MRS broth supplemented with 50% (*v*/*v*) glycerol. Cell morphology was observed using scanning electron microscopy (SEM).

Species identification was performed via 16S rRNA gene sequence analysis. Genomic DNA from the isolate was extracted using the Bacterial Genomic DNA Extraction Kit (Vazyme, Nanjing, China) following the manufacturer’s instructions. The 16S rRNA gene was amplified by polymerase chain reaction (PCR) using the universal primers 27F (5′-AGAGTTTGATCCTGGCTCAG-3′) and 1492R (5′-GGTTAACCTTGTTACGACT-3′). The PCR amplification protocol consisted of an initial denaturation at 94 °C for 5 min, followed by 30 cycles of denaturation at 94 °C for 30 s, annealing at 56 °C for 30 s, an extension at 72 °C for 1 min, and a final extension at 72 °C for 10 min. The amplified PCR products were sequenced by Anhui General Biotechnology Co., Ltd. (Chuzhou, China). The obtained sequences were compared against the NCBI database using BLAST (https://blast.ncbi.nlm.nih.gov/Blast.cgi) to determine the genus and species of the isolate. A phylogenetic tree was constructed using MEGA version 12.1 software with the neighbor-joining algorithm.

To confirm the species-level taxonomic assignment, Average Nucleotide Identity (ANI) was calculated between HI3 and reference *Enterococcus* genomes using a k-mer-based method (k = 25 and k = 30) with forward and reverse complement strand matching. Pairwise ANI values were calculated using the formula ANI = (S/M)^1/k^ × 100, where S is the number of shared k-mers, and M is the minimum of the query and reference k-mer counts. Digital DNA–DNA hybridization (dDDH) values were estimated using the GGDC Formula 2 approach. The species boundary thresholds of ANI ≥ 95% and dDDH ≥ 70% were applied [20,21].

### 2.2. Growth Curve Determination

The activated bacterial culture was inoculated (1%, *v*/*v*) into fresh MRS broth and incubated aerobically with shaking (200 rpm) at 37 °C for 24 h. Samples were aseptically collected every 2 h for the first 12 h and every 4 h for the subsequent 12 h. The optical density at 600 nm (OD_600_) was measured using an Infinite M Nano microplate reader (Tecan, Männedorf, Switzerland). The assay was performed with three biological replicates, and the growth curve was plotted based on the mean OD_600_ values over time.

### 2.3. Acid and Bile Salt Tolerance

To evaluate acid tolerance, the method described by Zhang et al. [22] and Safi et al. [23] was followed. An overnight culture of the freshly activated strain was inoculated (10%, *v*/*v*) into 10 mL of MRS broth. The pH of the medium was adjusted to different values (pH 2, 3, 5, and 7) using hydrochloric acid. The cultures were incubated aerobically with shaking (200 rpm) at 37 °C for 6 h. During incubation, samples were aseptically collected every 2 h in a laminar flow cabinet for OD_600_ measurement. The survival rate was calculated using the following formula [24]: survival rate (%) = (OD_600_ of the treated medium/OD_600_ of the untreated medium) × 100%.

For bile salt tolerance assessment, a freshly activated overnight culture was inoculated (10%, *v*/*v*) into 10 mL of MRS broth containing different concentrations of bile salts: 0%, 0.5%, 1%, and 2% (*w*/*v*). Each concentration was tested in triplicate. The cultures were incubated at 37 °C with shaking at 200 rpm for 6 h. OD_600_ was measured every 2 h, and the survival rate was calculated as described above. All assays were performed in triplicate.

### 2.4. Antimicrobial Susceptibility Testing

Antimicrobial susceptibility was evaluated using the Kirby-Bauer disk diffusion method [25]. Ten antibiotics spanning eight classes were tested, including penicillin, ampicillin, ceftriaxone, erythromycin, tetracycline, ciprofloxacin, chloramphenicol, co-trimoxazole (SXT), lincomycin, and gentamicin. Briefly, 100 µL of an overnight culture was spread onto MRS agar plates. Antibiotic disks were placed on the inoculated plates, which were then incubated at 37 °C for 16–24 h. The diameters of the inhibition zones were measured after incubation. Results were interpreted according to the guidelines provided by the Clinical and Laboratory Standards Institute (CLSI VET01S 2024) [26]. The specific breakpoints used for *Enterococcus* are presented in Table 1.

### 2.5. Hemolysis Assay

To evaluate the hemolytic activity of the bacterial strain, an overnight culture of HI3 incubated in MRS broth was streaked onto blood agar plates containing 5% sheep blood. The plates were then incubated at 37 °C for 48 h. After incubation, the plates were examined for the presence of clear zones (indicative of β-hemolysis), greenish zones (α-hemolysis), or no change (γ-hemolysis) around the bacterial colonies.

### 2.6. Whole-Genome Sequencing and Analysis

Genomic DNA of the strain was initially extracted using the Invitrogen PureLink^®^ Genomic DNA Kit (Thermo Fisher Scientific, Carlsbad, CA, USA). The quantity and quality of the extracted DNA were assessed using a NanoDrop ND-1000 spectrophotometer (Thermo Fisher Scientific (Waltham, MA, USA)). The DNA was further purified using the Quick-DNA Miniprep Plus Kit (Zymo Research, Irvine, CA, USA). A dual sequencing strategy was employed: Total genomic DNA was sequenced on the Illumina NovaSeq platform (Illumina, San Diego, CA, USA) (paired-end, 2 × 150 bp) and the Oxford Nanopore PromethION platform (Oxford Nanopore Technologies, Oxford, UK). Illumina sequencing generated 583.3 Mb of clean data (Q20 = 98.53%, Q30 = 97.27%), corresponding to approximately 191-fold genome coverage. Nanopore sequencing produced 169,062 reads totaling 1165 Mb (N50 = 11,793 bp), corresponding to approximately 381-fold coverage. Raw reads were subjected to quality control using Trimmomatic v0.36. Subsequently, a hybrid assembly integrating NGS and PacBio data was performed using Unicycler v0.5.0 to obtain a high-quality draft genome, which was then circularized using Circlator v1.5.5. Hybrid assembly using both short and long reads yielded four circularized replicons with zero gaps: one chromosome of 2,870,837 bp and three plasmids designated SX.P1 (116,676 bp), SX.P2 (42,557 bp), and SX.P3 (25,787 bp), for a total genome size of 3,055,857 bp with an overall GC content of 36.68%. Circularization of all four replicons was verified by terminal redundancy. GC-depth scatter analysis confirmed a single Poisson distribution, indicating the absence of contaminating sequences. Gene prediction of the assembled genome was conducted using GeneMark (http://topaz.gatech.edu/GeneMark/ (accessed on 16 December 2025)). Functional annotation of genes was performed by alignment against multiple databases, including the non-redundant (NR in NCBI) database, SwissProt (http://uniprot.org (accessed on 16 December 2025)), KEGG (http://www.genome.jp/kegg/ (accessed on 16 December 2025)), GO (http://www.geneontology.org/ (accessed on 16 December 2025)), COG (http://www.ncbi.nlm.nih.gov/COG (accessed on 16 December 2025)), and CAZy (http://www.cazy.org/ (accessed on 16 December 2025)). In addition, tRNAs were identified using the tRNAscan-SE (v2.0.4, http://lowelab.ucsc.edu/tRNAscan-SE (accessed on 16 December 2025)) and rRNAs were determined using the RNAmmer (v1.2, http://www.cbs.dtu.dk/services/RNAmmer/ (accessed on 16 December 2025)).

### 2.7. Genome Mining of Probiotic Genes

Functional annotation of the gene models identified by GeneMark (http://topaz.gatech.edu/GeneMark/ (accessed on 16 December 2025)) was performed using the blastp module against the KEGG database. In addition, genes responsible for probiotic characteristics in *Enterococcus*, such as those involved in acid tolerance, bile salt tolerance, heat and cold stress resistance, were retrieved from the functional annotation files based on previously reported studies [27,28,29,30].

### 2.8. Genome Mining of Antimicrobial Gene Clusters

Antimicrobial activity plays a crucial role in the ability of probiotics to inhibit gastrointestinal infections. With the increasing availability of whole-genome sequencing technologies and various genome mining tools, it is now feasible to predict antimicrobial compounds in silico [31]. AntiSMASH 5.0 (https://antismash.secondarymetabolites.org/ (accessed on 19 February 2026)) was employed to detect biosynthetic gene clusters of secondary metabolites in bacterial genomes, with default settings enabled for compound structure prediction, assembly line analysis, and regulatory elements identification [32,33]. Additionally, BAGEL 4.0 (http://bagel4.molgenrug.nl/ (accessed on 19 February 2026)) was utilized for comprehensive mining of biosynthetic gene clusters of ribosomally synthesized and post-translationally modified peptides (RiPPs) and bacteriocins from bacterial DNA sequences [34,35]. Both analyses were conducted using the default parameters provided by each platform.

### 2.9. Safety Assessment: Antibiotic Resistance and Virulence Factor Genes

To further evaluate the safety of strain HI3, antibiotic resistance genes and virulence factor genes were identified from its genome sequence. Gene models were predicted using GeneMark (http://topaz.gatech.edu/GeneMark/ (accessed on 16 December 2025)). For antibiotic resistance gene annotation, all predicted protein sequences were aligned against the Comprehensive Antibiotic Resistance Database (CARD, https://card.mcmaster.ca/ (accessed on 16 December 2025)) using BLASTp v2.9.0+ (E-value ≤ 1 × 10^−5^). Two complementary analyses were conducted. First, BLASTp hits were filtered using stringent criteria (identity ≥ 70%, coverage ≥ 90%) to identify high-confidence antibiotic resistance determinants. Second, to provide a comprehensive inventory of all CARD-detectable resistance-associated sequences, all BLASTp hits were retained without identity or coverage thresholds and manually classified into four categories based on replicon origin, percent identity, resistance mechanism, and mobile genetic element (MGE) context.

For virulence factor gene annotation, Diamond BLASTp was employed to search against the Virulence Factors of Pathogenic Bacteria (VFDB). The following parameters were used: an E-value cutoff of 1 × 10^−5^, minimum identity threshold of 70%, and minimum subject coverage of 90%. Genes showing significant hits to known resistance determinants or virulence factors were retrieved and manually curated to compile a list of putative antibiotic resistance and virulence factor genes in the HI3 genome.

To evaluate the mobility potential of the plasmid-borne tetracycline resistance genes, the complete nucleotide sequence of plasmid 3 (SX.P3, JBWQFC010000004.1, 25,787 bp) was extracted from the HI3 whole-genome assembly. All 23 coding sequences (CDSs) on plasmid 3 were manually curated, and functional annotation was refined by BLASTP searches against the NCBI non-redundant (NR) protein database and cross-referenced against the Comprehensive Antibiotic Resistance Database (CARD) and the ISFinder database for insertion sequence identification. Mobile genetic elements were classified into the following categories based on sequence homology and conserved domain architecture: insertion sequences and transposases; integrases and site-specific recombinases; resolvases; conjugative transfer proteins (tra family); and mobilization (Mob/Pre) relaxases. The genetic organization of the *tet(M)*-containing region was compared with the canonical architecture of Tn916/Tn1545 family conjugative transposons [36,37].

### 2.10. Statistical Analysis

All experiments were performed in triplicate. For acid tolerance and bile salt tolerance assays, differences among multiple groups at each time point were analyzed using SPSS version 27.0.1 statistical software (SPSS Inc., Chicago, IL, USA) by one-way analysis of variance (ANOVA). *p* < 0.05 was considered significant. Results are presented as mean ± standard deviation (SD).

## 3. Results

### 3.1. Identification of the Strain

**Figure 1:**
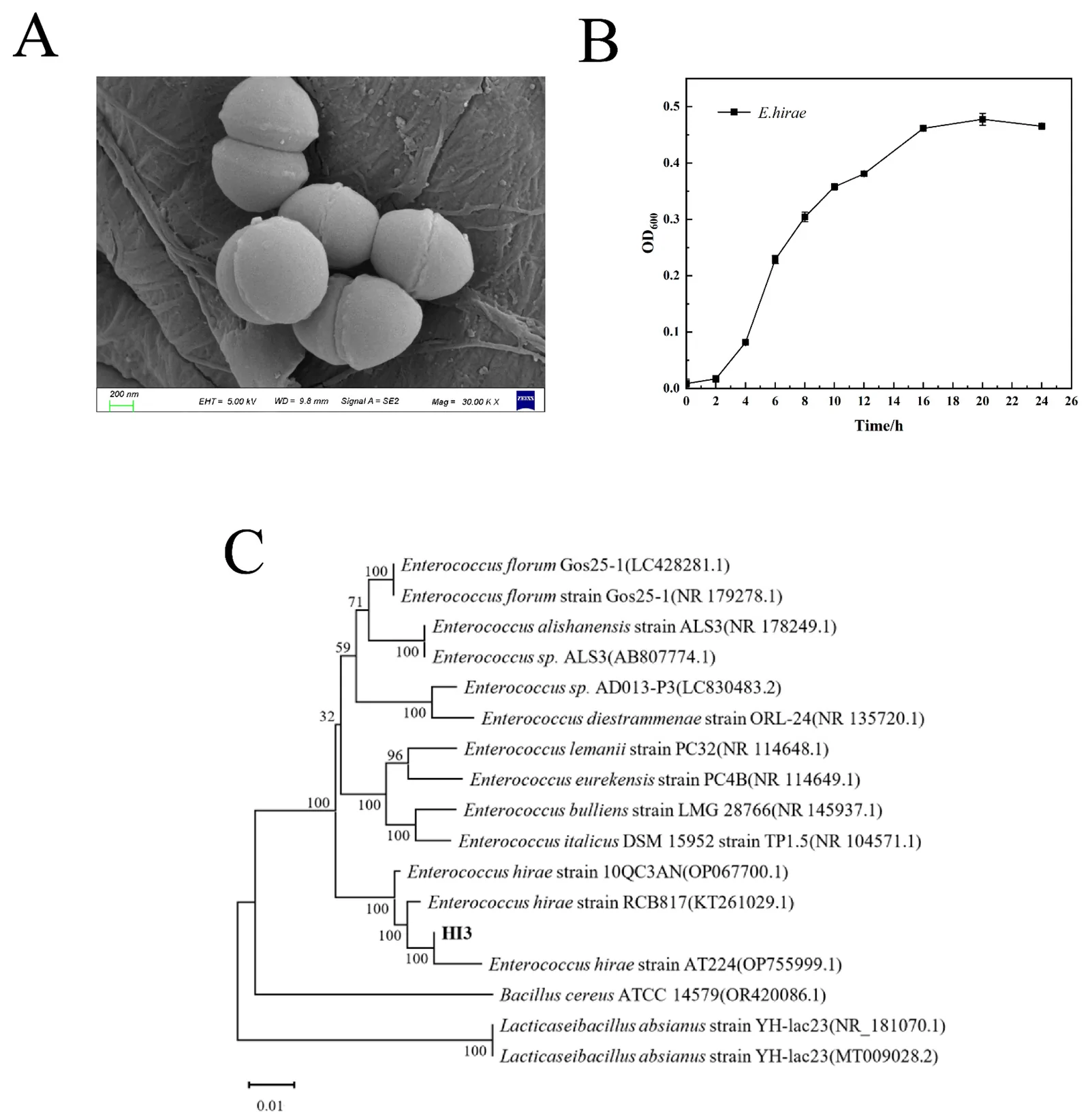
Scanning electron microscopy image (**A**), growth curve of *Enterococcus hirae* HI3 (**B**), and phylogenetic tree of strain HI3 based on 16S rRNA gene sequences (**C**).

Following isolation and cultivation, the strain typically formed small, grayish-white colonies on agar plates, which were circular, smooth, with entire margins, moist in texture, and non-pigmented. Observation under scanning electron microscopy (SEM) clearly revealed its typical coccoid morphology, with cells predominantly appearing spherical or ovoid, often arranged in pairs, short chains, or small clusters (Figure 1A). The HI3 strain, which exhibited 100% 16S rRNA gene sequence similarity with the *Enterococcus hirae* strain AT224 (OP755999.1), was identified as a strain of this species and named as *Enterococcus hirae* HI3 (Figure 1C). To further validate the species assignment at the genome level, Average Nucleotide Identity (ANI) and digital DNA–DNA hybridization (dDDH) analyses were performed using the whole-genome sequence data. Pairwise ANI analysis revealed 98.92% and 98.00% identity with the complete *E. hirae* genomes 10K2I (GCF_056858135.1) and EM2 (GCF_054952805.1), respectively, both well above the 95% species boundary threshold (Table 2). In contrast, ANI values with other *Enterococcus* species ranged from 70.33% to 81.41%, confirming that HI3 is distinct from *E. faecalis*, *E. faecium*, *E. avium*, and *E. mundtii*. The dDDH estimates were consistent with same-species assignment, though the k-mer-based dDDH approach may yield conservative estimates compared to BLAST-based methods (e.g., GGDC web server).

### 3.2. Growth Characteristics of HI3

The growth curve of HI3 is shown in Figure 1B. During the initial 0–2 h of cultivation, the growth rate of the strain was relatively slow. Subsequently, from 2 to 8 h, strain HI3 entered the exponential growth phase, exhibiting an exponential growth pattern characterized by a marked increase in growth rate. From 8 to 20 h, the growth rate showed little increase, and after 20 h, the OD_600_ value of the strain exhibited a declining trend.

### 3.3. Acid and Bile Salt Tolerance of HI3

**Figure 2:**
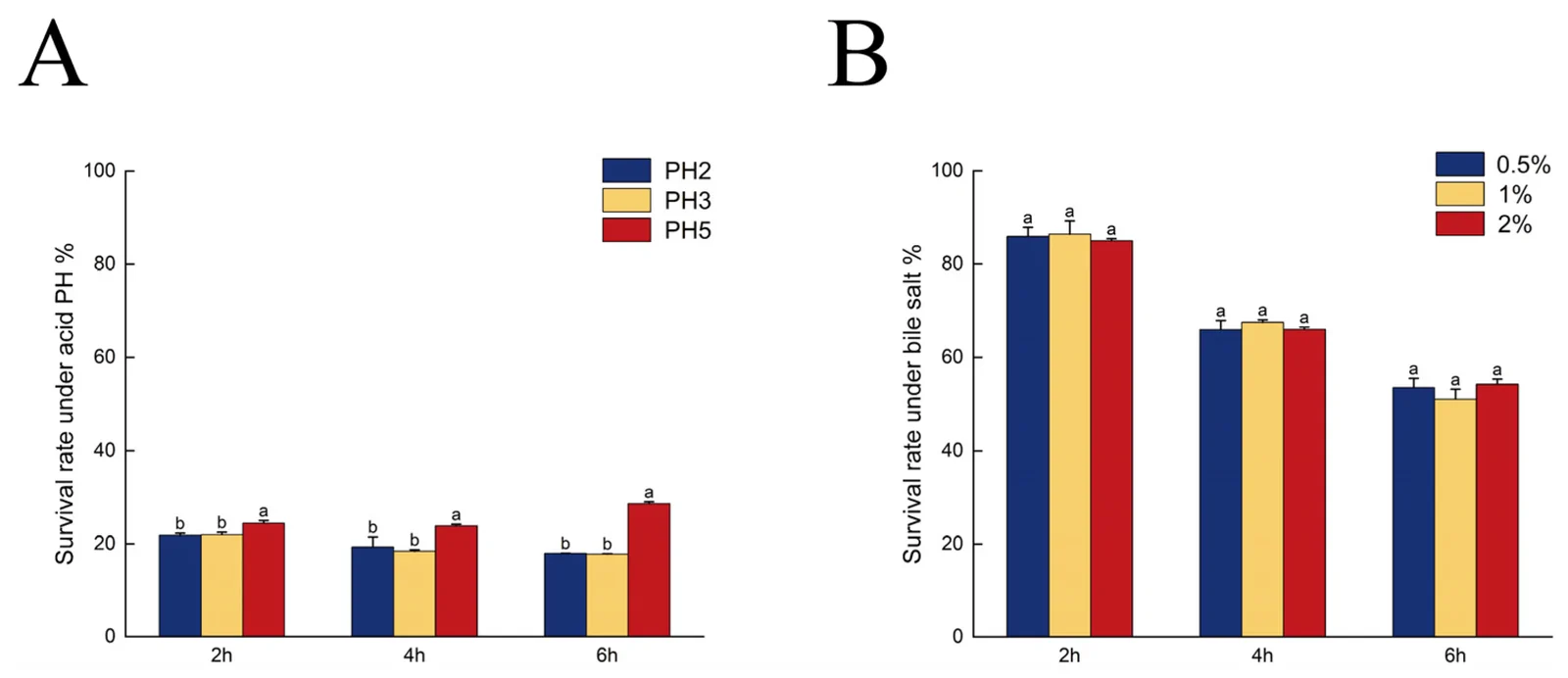
Survival rates of *Enterococcus hirae* HI3 at different pH values (**A**) and under different bile salt concentrations (**B**). Different lowercase letters (a, b) above the bars indicate significant differences at the same time point (*p* < 0.05).

The acid and bile salt tolerance results showed that strain HI3 maintained viability under high acidity and bile salt concentrations (Figure 2A,B). After 4 h of incubation at pH 2, pH 3, and pH 5, the survival rates of HI3 were 19.31% (95% CI: 15.83–22.78%), 18.40% (95% CI: 17.87–18.94%), and 23.84% (95% CI: 23.28–24.41%), respectively; after 6 h, the survival rates were 17.97% (95% CI: 17.78–18.16%), 17.78% (95% CI: 17.56–17.99%), and 28.61% (95% CI: 27.99–29.23%), respectively. Notably, at each time point (2 h, 4 h, and 6 h), the survival rate of the strain at pH 5 was significantly higher than that at pH 2 and pH 3 (*p* < 0.05). In the bile salt tolerance assay, after 6 h of growth in the presence of 0.5%, 1%, and 2% bile salts, the survival rates were 53.49 (95% CI: 48.55–58.43%), 51.01 (95% CI: 47.63–54.39%), and 54.22 (95% CI: 52.45–55.98%), respectively. No significant differences were observed among the different bile salt concentration groups at any of the time points examined.

### 3.4. Antimicrobial Susceptibility Testing Results

The results of antimicrobial susceptibility testing are presented in Table 3, revealing the susceptibility profiles of strain HI3 to a panel of antibiotics. Specifically, the inhibition zone diameters were as follows: penicillin (PEN) 27.17 ± 2.25 mm, ampicillin (AMP) 28.17 ± 1.26 mm, erythromycin (E) 30.5 ± 0.87 mm, chloramphenicol (C) 31.17 ± 1.89 mm, ceftriaxone (CTR) 16.17 ± 0.58 mm, and ciprofloxacin (CIP) 17.67 ± 0.58 mm. No inhibition zones were observed for tetracycline (TET), sulfamethoxazole/trimethoprim (SXT), lincomycin (MY), and gentamicin (GEN), with zone diameters equal to the disk diameter (6.0 mm), indicating phenotypic resistance to these four antibiotics.

### 3.5. Hemolysis Activity

**Figure 3:**
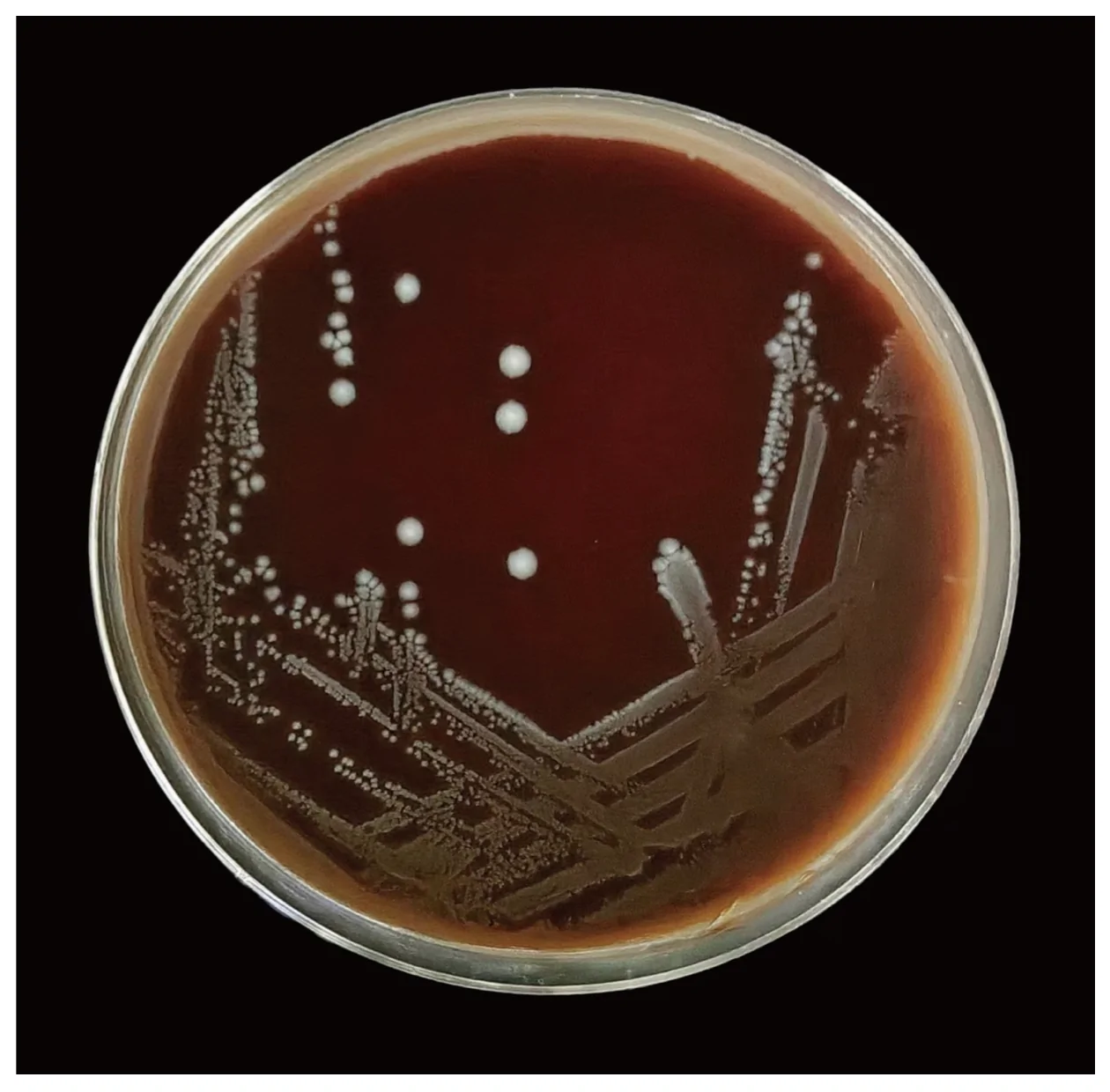
Hemolytic activity of strain HI3 on Columbia blood agar containing 5% sheep blood after 48 h of incubation. No hemolytic zone (γ-hemolysis) was observed around the bacterial colonies.

After 48 h of incubation on Columbia blood agar containing 5% sheep blood, HI3 exhibited no zone of hemolysis (γ-hemolysis; Figure 3).

### 3.6. Genomic Features of HI3

**Figure 4:**
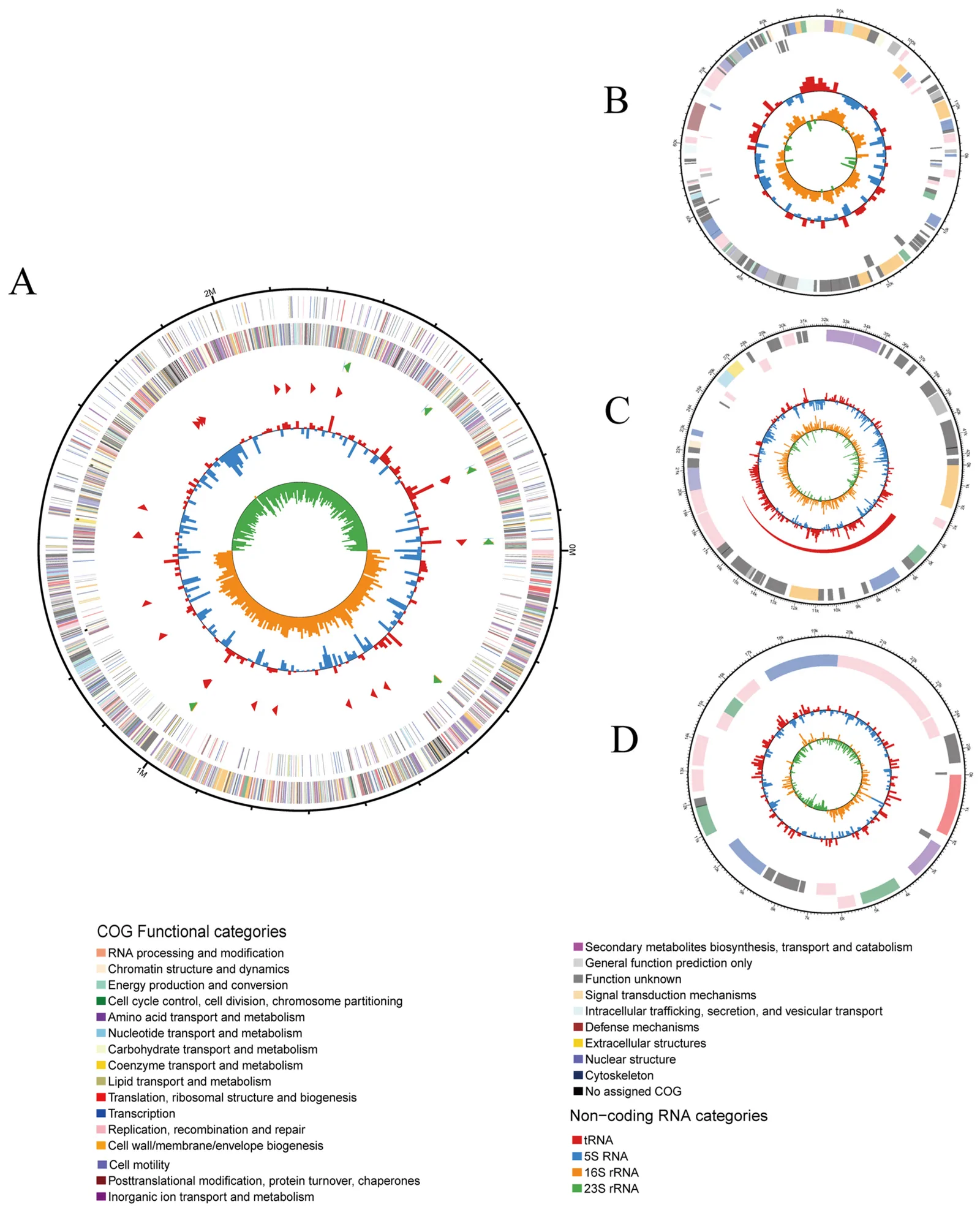
Genomic circle map of strain HI3, showing the chromosome (**A**) and plasmids (**B**–**D**). From the outside to the inside, the first circle indicates the genome size; the second and third circles represent coding sequences CDS on the positive and negative strands, respectively, with different colors indicating different COG functional categories; the fourth circle shows rRNA and tRNA; the fifth circle represents GC content, with outward red peaks indicating regions where the GC content is higher than the genome-wide average (higher peaks indicating greater deviation), and inward blue peaks indicating regions where the GC content is lower than the genome-wide average; the innermost circle shows the GC skew value, calculated as (G − C)/(G + C).

Whole-genome sequencing of HI3 revealed that the genome of this strain consists of one circular chromosome (Figure 4A) and three circular plasmids (Figure 4B–D). The total genome size is 3,055,857 bp, with an N50 of 2,870,837 bp and a GC content of 36.68%. A total of 3061 genes were predicted, including 71 tRNA and 18 rRNA genes. In addition to the two intact prophage regions detected, other genomic features of HI3, including CRISPR regions, ARGs, and VFGs, are summarized in Table 4.

### 3.7. COG Functional Annotation and Classification

**Figure 5:**
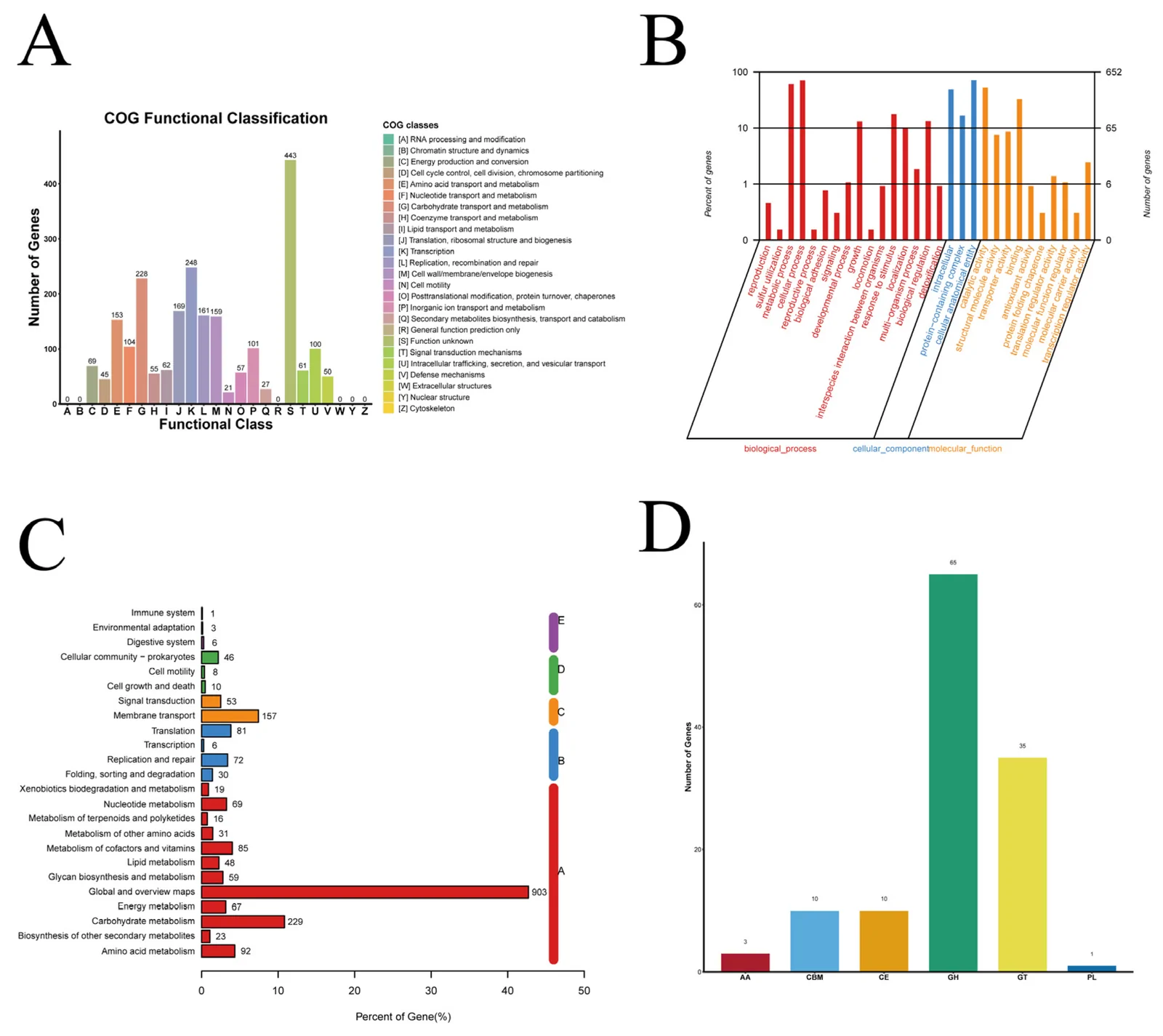
COG functional annotation results (**A**), GO database annotation results (**B**), KEGG functional annotation results (**C**), and CAZy annotation statistics (**D**) of the HI3 genome. Note: metabolism (A), genetic information processing (B), environmental information processing (C), cellular processes (D), and organismal systems (E).

Using eggnog-mapper (v5.0) for alignment against the COG database, we functionally annotated 2184 genes. Based on protein evolutionary relationships, these genes were classified into 19 categories. Among these, 248 genes were involved in transcription, 228 genes in carbohydrate transport and metabolism, and 169 genes in translation, ribosomal structure, and biogenesis (Figure 5A).

### 3.8. GO Database Annotation and Classification

Gene Ontology (GO) analysis revealed that a total of 652 genes in HI3 were functionally annotated, covering the three categories of cellular component, molecular function, and biological process. A total of 394 genes were annotated to cellular component terms, with the majority of annotations associated with cellular structural entities and intracellular functions. In terms of molecular function, 392 genes were annotated, with catalytic activity and binding representing the predominant functions. For the biological process, a total of 412 genes were annotated, with cellular and metabolic processes being the major functional categories (Figure 5B).

### 3.9. KEGG Functional Annotation and Classification

The KEGG (Kyoto Encyclopedia of Genes and Genomes) analysis revealed that a total of 1400 genes were annotated, covering five major categories: Metabolism, Genetic Information Processing, Environmental Information Processing, Cellular Processes, and Organismal Systems. Among these, the Metabolism category had the highest number of annotated genes, with carbohydrate metabolism being particularly abundant, accounting for 229 genes, followed by amino acid metabolism (92 genes), and metabolism of cofactors and vitamins (85 genes). In addition, a substantial number of genes were involved in Environmental Information Processing, especially membrane transport, with 157 genes annotated in this subcategory (Figure 5C).

### 3.10. CAZy Functional Annotation and Classification

Analysis using the CAZy database revealed that strain HI3 harbors 65 genes annotated as glycoside hydrolases, 35 genes as glycosyltransferases, 10 genes as carbohydrate esterases, 10 genes as carbohydrate-binding modules, 3 genes as auxiliary activity enzymes, and 1 gene as polysaccharide lyase (Figure 5D).

### 3.11. Predicted Probiotic-Associated Genes in the HI3 Genome

Genomic annotation of HI3 revealed at least 43 genes associated with probiotic characteristics, including genes involved in bile salt (e.g., *cbh*, *hupB*), acid (e.g., *atpA–G*, *nhaC*), temperature (e.g., *clp* family, *groEL*, *dnaK*, *cspA*), and oxidative stress (e.g., *sod2*, *trxA*) tolerance, as well as adhesion (e.g., *ltaS*, *srtA*, *eno*) (Table 5).

### 3.12. Gene Cluster Analysis of Antimicrobial Compounds

**Figure 6:**
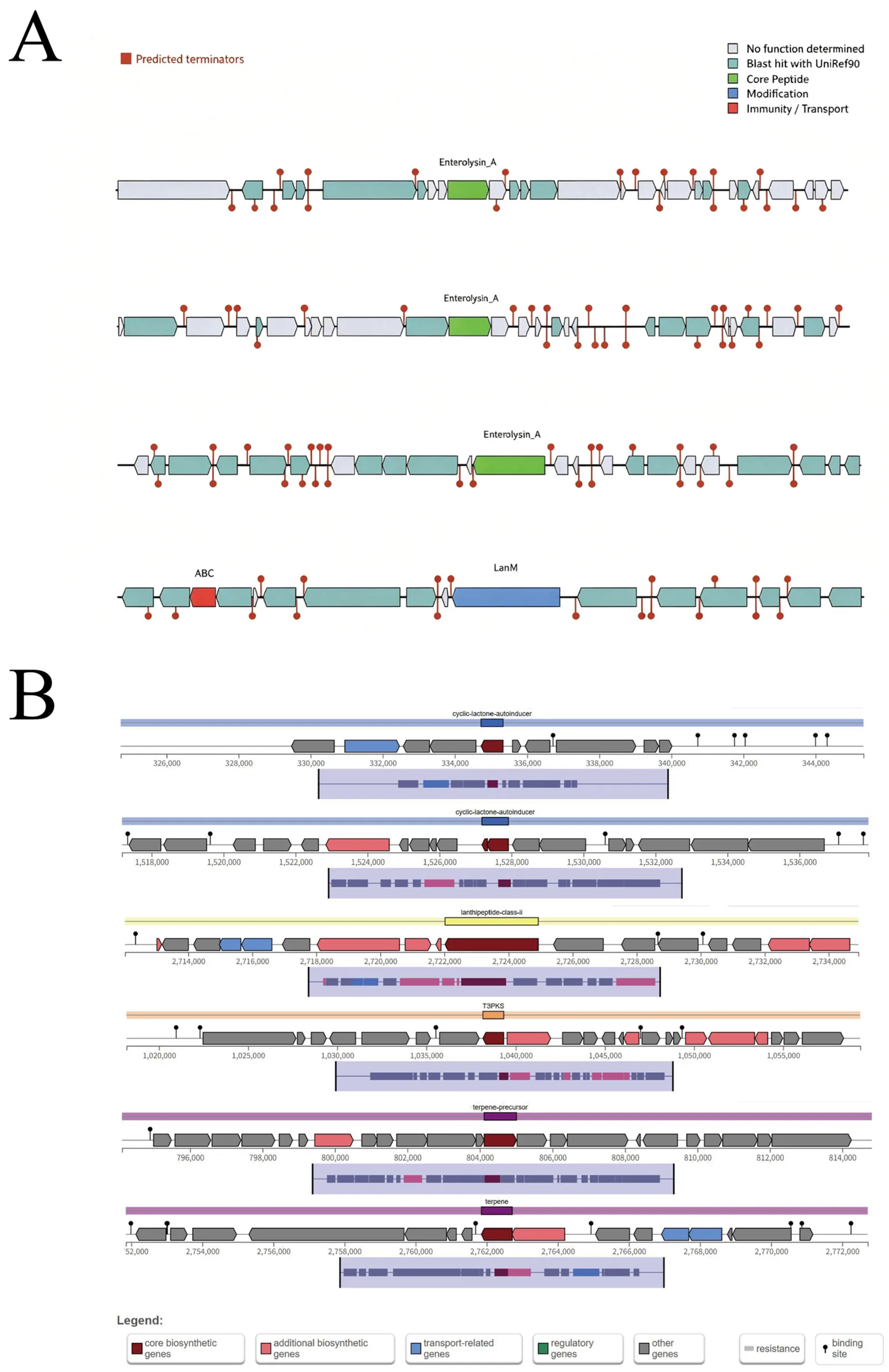
Gene clusters of antimicrobial peptides predicted using the online servers BAGEL4 (**A**) and antiSMASH 5.0 (**B**).

The BAGEL4.0 database revealed the presence of four key gene clusters predicted to be involved in bacteriocin synthesis and secretion, among which three were associated with the biosynthesis of the bacteriocin Enterolysin A, and one was associated with the synthesis of the core peptide class II lanthipeptide (Figure 6A). Using antiSMASH 5.0 to predict secondary metabolite biosynthetic gene clusters in the HI3 genome, we identified six gene clusters in our strain, which were associated with the biosynthesis of a cyclic lactone autoinducer, a terpene precursor, T3PKS, and a class II lanthipeptide (Figure 6B).

### 3.13. Identification of Antibiotic Resistance Genes and Virulence Factor Genes

CARD BLASTp analysis of the HI3 genome identified 87 resistance-associated sequences (E-value ≤ 1 × 10^−5^, Supplementary Table S1). After applying stringent filtering criteria (identity ≥ 70%, coverage ≥ 90%), 16 high-confidence hits were retained (Table 6). These 16 hits comprised two acquired tetracycline resistance genes, *tet(M)* and *tet(L)*, both carried on plasmid 3. One hit corresponded to *AAC(6′)-Iid* (SX002280; 97.8% identity), a chromosomally encoded aminoglycoside acetyltransferase that represents a conserved intrinsic trait of *E. hirae*. Nine hits were chromosomal housekeeping genes (*EF-Tu*, *rpoB*, *rpoC*, *fusA*, *rpsE*, *D-Ala-D-Ala ligase*, and others) whose CARD matches reflect mutation-associated resistance variants from other bacterial species rather than acquired resistance in HI3. The remaining four hits were chromosomal efflux-related loci (*liaFSR*, *efrA*). No high-confidence matches were found for acquired resistance to clinically important antibiotics such as vancomycin, β-lactams, linezolid, or daptomycin.

Plasmid 3 is a 25,787 bp circular plasmid containing 23 predicted CDSs (Supplementary Table S2). Analysis of these CDSs identified ten mobile genetic element (MGE)-associated genes. The *tet(M)* and *tet(L)* genes are located on the positive strand, separated by a single hypothetical protein-encoding sequence. Notably, a conjugal transfer protein gene was identified 376 bp upstream of *tet(M),* and a Mob/Pre family relaxase gene (encoding a plasmid recombination enzyme) was identified 584 bp downstream of *tet(L)*. Three identical copies of IS1216 and an IS30-like transposase (IS1252 family) were distributed across the plasmid, together with a resolvase and an integrase. No canonical type IV secretion system components or a complete tra operon were detected, suggesting that plasmid 3 harbors genetic features consistent with mobilizable plasmids. However, functional transferability remains to be experimentally demonstrated.

Additionally, 20 genes related to virulence factors were identified. The HI3 genome harbored genes encoding the endocarditis-specific antigen (*efaA*), bile salt hydrolase (*bsh*), chaperonin GroEL (*groEL*), elongation factor Tu (*tufA*), glyceraldehyde-3-phosphate dehydrogenase (*plr*/*gapA*), phosphopyruvate hydratase (*eno*), ATP-dependent Clp protease (*clpP*), two-component response regulator (*lisR*), and multiple genes involved in polysaccharide and lipoteichoic acid biosynthesis (e.g., *cpsA*/ *uppS*, *cpsB*/*cdsA*, *galU*, *rfbA*, *rfbB*, *gndA*, SGO_RS09900, SPD_RS01770, *bopD*, EFMU0317_RS16950) (Table 7). Notably, the major enterococcal virulence factors (e.g., *gelE*, *cyl*, *esp*, *hyl*) were not detected in the HI3 genome.

Among the 20 VFDB hits, five moonlighting proteins were identified: elongation factor Tu (*tufA*), enolase (*eno*), glyceraldehyde-3-phosphate dehydrogenase (*gapA*), chaperonin GroEL (*groEL*), and the Ace/Acm-family adhesin EfaA. Systematic comparison of these five proteins against the NR database revealed that all share 99–100% amino acid identity with orthologs from non-pathogenic *Enterococcus* species, including *E. hirae* and *E. villorum*, and none matched pathogenic *E. faecalis* or *E. faecium* as their top hit (Supplementary Table S3). This pattern is consistent with the genome-wide NR annotation, in which 95.9% of HI3 genes have top hits to *Enterococcus* species, dominated by *E. hirae* (82.2%). However, it is important to note that high sequence similarity to orthologs from non-pathogenic species does not, by itself, exclude the potential for virulence-related functions, particularly for moonlighting proteins whose pathogenic roles may depend on expression level, cellular context, or host environment rather than primary sequence alone. Therefore, these findings should be interpreted as supportive of a commensal-like genomic background, but not as definitive evidence of non-virulence.

## 4. Discussion

This study provides an integrated phenotypic and genomic characterization of *Enterococcus hirae* HI3, a strain isolated from the jejunum of healthy Hu sheep. The combined results indicate that HI3 possesses key traits compatible with probiotic functionality, including gastrointestinal stress tolerance, antibiotic susceptibility profile, and a diverse repertoire of genes encoding probiotic-associated functions and antimicrobial metabolites.

Resistance to gastric acidity and intestinal bile salts is a prerequisite for probiotic efficacy [60]. HI3 exhibited moderate acid tolerance, with a survival rate of approximately 18.40% at pH 3 after 4 h of incubation, indicating a considerable loss of viability under acidic conditions. At pH 5, the strain maintained higher survival (28.61% after 6 h), suggesting that it may better withstand the less severe acidic environment encountered after gastric emptying. Compared with other studies using similar OD_600_-based methods, HI3 showed relatively low acid tolerance. For instance, Li et al. [61] reported 53.99% survival for an *E. hirae* strain from wild boar gut after 4 h at pH 3, and Zhang et al. [22] reported approximately 68% survival for four *Lactobacillus* strains under the same conditions, although direct comparisons should be made with caution due to strain-specific variability. Furthermore, HI3 maintained > 50% survival after 6 h of exposure to all tested bile salt concentrations (0.5–2%), with no significant concentration-dependent effect, indicating robust and constitutive bile salt tolerance. Consistent with our results, numerous studies have shown that enterococci can survive under low pH and high bile salt conditions [62,63,64,65]. These characteristics are highly consistent with the genomic identification of multiple acid tolerance-related genes (*atpA–G*, *nhaC*) and bile salt tolerance-related genes (*cbh*, *hupB*) in the genome. For instance, the identified *atpA–G* and *cbh* genes encode the F0F1 ATP synthase and choloylglycine hydrolase, respectively. The F0F1 ATP synthase helps bacteria survive under acidic conditions by consuming ATP to pump excess intracellular hydrogen ions (H^+^) out of the cell, thereby maintaining a near-neutral cytoplasmic pH environment [66]. Choloylglycine hydrolase is a multifunctional bile salt hydrolase that primarily functions by hydrolyzing the amide bonds of conjugated bile acids, thereby reducing their antimicrobial toxicity and facilitating bacterial adaptation to the intestinal environment [67]. These genes collectively enhance tolerance to acidic and bile-salt conditions, supporting the survival of HI3 in the gastrointestinal tract.

Antimicrobial susceptibility testing revealed that HI3 exhibits certain advantages compared with other previously reported probiotic strains. For instance, HI3 remained susceptible to penicillin, ampicillin, erythromycin, and chloramphenicol, whereas *Enterococcus faecium* BS5 was reported to be resistant to these agents [68], and *Enterococcus faecalis* Spp19 exhibited resistance to penicillin and ampicillin [69]. Susceptibility to these first-line anti-enterococcal agents is clinically desirable, as it ensures that the probiotic can be eliminated by conventional antibiotics if necessary. Additionally, the resistance of HI3 to tetracycline and co-trimoxazole (SXT) is consistent with the findings reported for *Enterococcus hirae* SMB16, a potential probiotic strain isolated from goat and sheep milk [63].

A total of 16 putative antibiotic resistance genes were identified in the HI3 genome. Notably, no acquired resistance genes for β-lactams (e.g., *blaZ*, altered *pbp5*), macrolides (e.g., *erm* family), or glycopeptides (e.g., *van* gene clusters) were detected. The absence of β-lactamase genes and macrolide resistance determinants is consistent with the observed susceptibility to penicillin, ampicillin, and erythromycin. Although vancomycin susceptibility was not tested in this study, the lack of van genes is a favorable safety indicator for a probiotic candidate. For ceftriaxone, although a zone of inhibition was observed, this does not indicate true susceptibility. According to CLSI VET01S, cephalosporins may appear active *in vitro* against enterococci but are clinically ineffective due to intrinsically low-affinity penicillin-binding proteins (PBP5/PBP4) that mediate resistance at physiological concentrations. For co-trimoxazole (SXT), phenotypic resistance without detectable genes is consistent with the intrinsic resistance of enterococci, which utilize exogenous folates to bypass the drug-targeted folate synthesis pathway [70]. For lincomycin, resistance in the absence of acquired erm genes is explained by the natural expression of *lsa* genes encoding ABC transporters responsible for drug efflux [71]; indeed, *lsa* genes were detected in the HI3 genome. For gentamicin, resistance was observed despite the presence of only *AAC(6′)-Iid*; genes conferring high-level gentamicin resistance (*aph(2″)-Ib*, *aph(2″)-Id*, and *aac(6′)-Ie-aph(2″)-Ia*) were absent [72]. The presence of *AAC(6′)-Iid* may account for the low-level gentamicin resistance observed in the disk diffusion assay. For ciprofloxacin, the intermediate phenotype may be partially explained by *efrA* and *efrB*, encoding the EfrAB multidrug efflux pump, which transports fluoroquinolones and macrolides [73]. However, HI3 remained susceptible to erythromycin, indicating that the EfrAB pump does not confer clinically relevant macrolide resistance in this strain. The tetracycline resistance phenotype corresponds to the presence of *tet(M)* and *tet(L)*, both of which were located on plasmid 3.

The close association of *tet(M)* and *tet(L)* with a conjugal transfer protein, a Mob/Pre relaxase, IS1216 elements, and an integrase on plasmid 3 is consistent with the modular architecture of Tn916/Tn1545-type conjugative transposons, which are well-documented vectors for tetracycline resistance dissemination in Gram-positive bacteria [37,74]. The presence of a relaxase further implies that plasmid 3 is mobilizable via complementation by helper plasmids [75]. While the bioinformatic evidence reveals a genetic context consistent with mobilization, it does not demonstrate functional transferability. Key experiments required to establish the transferability of *tet(M)* and *tet(L)* include filter-mating conjugation assays with suitable recipient strains, plasmid curing to confirm the exclusive plasmid location of these resistance determinants, and *in vitro* mobilization assays to test the functionality of the identified Mob/Pre relaxase and conjugal transfer protein. From a safety perspective, susceptibility to first-line anti-enterococcal drugs such as penicillin and ampicillin is a favorable feature, as it ensures that the probiotic can be effectively eliminated by conventional antibiotics if clinically necessary [76,77]. Additionally, the hemolysis assay revealed no hemolytic activity (γ-hemolysis), and major enterococcal virulence genes such as *gelE*, *cyl*, *esp*, and *hyl* were absent from the genome [78,79]. However, as noted in the subsequent sections, the absence of these classical virulence genes does not, on its own, constitute definitive evidence of safety, given the complexity of enterococcal pathogenicity and the presence of other VFDB-annotated genes requiring nuanced interpretation.

Whole-genome sequencing of HI3 provided a comprehensive genomic basis for evaluating its probiotic potential and safety [80]. The genome assembly (3.06 Mb, 36.68% GC) is consistent with other *E. hirae* isolates [81,82]. COG, GO, and KEGG functional annotations indicated that HI3 possesses a strong metabolic capacity, particularly in carbohydrate utilization (229 genes) and membrane transport (157 genes), consistent with adaptation to the intestinal environment [83,84]. CAZy database annotation revealed that HI3 harbors 65 glycoside hydrolase genes. These enzymes have the potential to degrade insoluble carbohydrates in the intestine, which could lead to the production of short-chain fatty acids (SCFAs), such as acetate, propionate, and butyrate [85,86]. SCFAs are known to modulate the gut microbiota, promote intestinal mucosal development, and facilitate nutrient absorption [87,88], representing a pathway through which probiotics may exert beneficial functions. However, the presence of glycoside hydrolase genes does not confirm actual enzyme activity or SCFA production *in vivo*. Functional assays, including enzymatic activity measurements and SCFA quantification, are required to validate this predicted metabolic capacity.

The antimicrobial activity of probiotics is central to their ability to inhibit intestinal pathogens and maintain gut microbiota homeostasis, with the synthesis of antimicrobial substances such as bacteriocins serving as the primary mechanism underlying this function [33]. Genomic analysis revealed that HI3 harbors four key gene clusters predicted to be involved in bacteriocin synthesis and secretion (three associated with Enterolysin A and one with class II lanthipeptide), as well as six secondary metabolite biosynthetic gene clusters. The presence of these gene clusters is consistent with the findings detected in the potential probiotic *Enterococcus hirae* by Li et al. [13]. The presence of these gene clusters suggests the genetic potential for antimicrobial peptide production, but this remains a prediction that requires experimental validation.

Enterolysin A, a cell wall-degrading bacteriocin, has been reported to exhibit activity against Gram-positive pathogens, including *Listeria* and *Staphylococcus* [89,90]. Class II lanthipeptides are ribosomally synthesized and post-translationally modified peptides with membrane-disrupting activity [91].

Beyond bacteriocins, six secondary metabolite clusters were identified, including cyclic-lactone-autoinducer (quorum sensing), terpene-precursor (predicted antioxidant/anti-inflammatory), and type III polyketide synthase (T3PKS, associated with polyketide metabolites) [92,93,94]. These clusters have been identified in other enterococci, supporting the genetic potential of this genus for secondary metabolite production [95,96]. However, it should be noted that the identification of biosynthetic gene clusters is based on genomic prediction and does not guarantee functional metabolite production. *In vitro* inhibition assays against specific enteric pathogens, as well as transcriptional and metabolomic validation of metabolite production, are required to confirm the antimicrobial capacity of HI3.

The HI3 genome also harbors genes potentially associated with probiotic traits, such as *grpE* (stress response), *dnaK*/*dnaJ* (DNA repair and stress adaptation), adhesion-related genes (*ltaS*, *srtA*, *eno*) [97,98,99], and EPS biosynthesis genes (*epsA*/*epsB*). However, experimental validation of their expression is required and will be addressed in our subsequent work.

The safety assessment in this study was guided by the EFSA FEEDAP Panel guidance on the characterization of microorganisms used as feed additives [100] and the FAO/WHO Guidelines for the Evaluation of Probiotics in Food [101]. These frameworks recommend the evaluation of antimicrobial susceptibility, the absence of acquired antimicrobial resistance genes of clinical relevance, and screening for known virulence determinants. In line with these frameworks, our study employed WGS-based screening of antimicrobial resistance genes (CARD database) and virulence factors (VFDB database) with phenotypic testing for antibiotic susceptibility and hemolytic activity. The HI3 genome harbors one CRISPR-Cas system and two intact prophage regions. Major enterococcal virulence determinants, including *gelE*, *cyl*, and *hyl*, were absent from the genome.

VFDB analysis identified 20 putative virulence-associated genes, the majority of which encode housekeeping or moonlighting proteins. Systematic NR database comparison showed that proteins such as *tufA*, *groEL*, *eno*, and *clpP* share 99–100% amino acid identity with orthologs from commensal *Enterococcus* species. While this high sequence conservation is consistent with their primary roles in essential cellular processes, it does not exclude the possibility that these proteins could acquire or exhibit virulence-related functions under specific conditions, as moonlighting activities are context-dependent and not reliably predicted by sequence homology alone. Regarding *efaA*, its presence requires cautious interpretation: although it has been linked to pathogenicity in some clinical isolates, it has also been identified in probiotic strains without associated disease [102,103,104]. The HI3 *efaA* allele shares 99.1% identity with the *E. hirae* ortholog and only 71–80% identity with *E. faecalis* and *E. faecium* clinical isolates, suggesting species-level conservation rather than virulence-associated adaptation. Nevertheless, the potential biological relevance of these VFDB-annotated genes cannot be fully resolved by genomic analysis alone. Notably, major enterococcal virulence genes (*gelE*, *cyl*, *esp*, *hyl*) were absent [77], and no hemolytic activity was detected. In summary, no known transferable virulence factors were identified in the HI3 genome, and no hemolytic activity was observed. While these findings are favorable at the screening level, they do not constitute definitive evidence of safety. Functional assays, including cytotoxicity testing, epithelial adhesion/invasion assays, and *in vivo* pathogenicity evaluation, are required before HI3 can be considered a probiotic candidate.

Based on phenotypic and genomic analyses, we have identified *Enterococcus hirae* HI3 as a preliminary probiotic candidate. However, certain limitations of the current study should be acknowledged. First, although we have performed a comprehensive *in vitro* characterization, including acid and bile salt tolerance based on OD_600_ measurements, which do not distinguish viable from non-viable cells, antibiotic susceptibility, hemolytic activity, and genome-based screening for antibiotic resistance genes and virulence factors, several key assays required to substantiate probiotic functionality were not conducted. These include adhesion to intestinal epithelial cells, antimicrobial activity against pathogens, autoaggregation, coaggregation, biofilm formation, antioxidant activity, immunomodulatory effects, and colonization potential. Therefore, the probiotic properties of HI3 remain to be experimentally validated. Second, although we performed a detailed bioinformatic analysis of plasmid 3 and identified a genetic context consistent with mobilization (including a conjugal transfer protein, a Mob/Pre relaxase, IS1216 elements, and an integrase) and confirmed the absence of a complete type IV secretion system, the functional transferability of the tetracycline resistance genes *tet(M)* and *tet(L)* has not been experimentally demonstrated. Conjugation experiments are required to assess whether this plasmid can be mobilized under relevant conditions before any field application of HI3 as a probiotic. Third, we did not perform minimum inhibitory concentration (MIC) determinations, which are the preferred method for distinguishing acquired from intrinsic resistance under EFSA guidance. The disk diffusion method used here provides a useful screening-level indication but is less precise than MIC-based approaches. Fourth, biofilm-forming capacity and comprehensive phenotypic virulence characterization (e.g., cytotoxicity assays, adhesion/invasion tests in epithelial cell lines) should be evaluated in future studies to provide a more complete safety profile for HI3. Last but not least, the safety and probiotic efficacy of HI3 have not yet been evaluated *in vivo*. Future studies should include feeding trials in ruminants to validate intestinal colonization capacity, modulatory effects on the host gut microbiota, and growth-promoting efficacy [105]. Direct evidence of antimicrobial activity against specific enteric pathogens also remains to be established, either through *in vitro* inhibition assays (e.g., agar well diffusion or co-culture methods) or *in vivo* pathogen-challenge experiments. Such investigations are essential for fully realizing the potential of *E. hirae* HI3 as a targeted probiotic for application in animal husbandry.

## 5. Conclusions

In this study, a strain of *Enterococcus hirae* HI3 was successfully isolated and identified from the intestine of Hu sheep. This strain exhibited robust growth capacity, moderate acid tolerance, and favorable bile salt tolerance, as well as susceptibility to penicillin, ampicillin, erythromycin, and chloramphenicol. No hemolytic activity was detected. Whole-genome analysis revealed that HI3 harbors multiple functional genes associated with carbohydrate metabolism, stress response, adhesion, and antimicrobial compound synthesis, as well as several bacteriocin biosynthetic gene clusters. The plasmid-borne tetracycline resistance genes *tet(M)* and *tet(L)* warrant further investigation to assess their transferability. The identification of several bacteriocin biosynthetic gene clusters within its genome further supports its potential antimicrobial activity. Taken together, the integrated phenotypic and genomic findings provide an initial characterization of HI3 and identify it as a strain deserving further investigation for potential probiotic application in ruminant production. Future studies should validate its probiotic functionality through *in vivo* trials and comprehensive safety assessments.

## Figures and Tables

**Table 1:** Breakpoints for antimicrobial susceptibility testing of *Enterococcus* spp.

Antibiotic Type	Antibiotics	Disk Content of Antimicrobials (μg/Disk)	Inhibition Zone Diameter Criteria (mm)		
			Resistant (R)	Intermediate (I)	Susceptible (S)
Beta-lactams	Penicillin (PEN)	10	≤14	-	≥15
	Ampicillin (AMP)	10	≤16	-	≥17
	Ceftriaxone (CTR)	30	-	-	-
Macrolides	Erythromycin (E)	15	≤13	14–22	≥23
Tetracycline	Tetracycline (TET)	30	≤14	15–18	≥19
Fluoroquinolones	Ciprofloxacin (CIP)	5	≤15	16–20	≥21
Phenylpropanoids	Chloramphenicol (C)	30	≤12	13–17	≥18
Sulfonamides	Co-trimoxazole (SXT)	25	-	-	-
Lincomycins	Lincomycin (MY)	2	-	-	-
Aminoglycosides	Gentamicin (GEN)	10	-	-	-

**Table 2:** Genome-based taxonomic validation of *Enterococcus hirae* HI3 using Average Nucleotide Identity (ANI) and digital DNA–DNA Hybridization (dDDH).

Reference Strain	Assembly	Size (Mb)	GC (%)	ANI (%)	dDDH (%)	Assignment
*E. hirae* 10K2I (GCF_056858135.1)	Complete	2.75	37.0	98.92	68.96	*E. hirae* (confirmed)
*E. hirae* EM2 (GCF_054952805.1)	Near-complete	2.95	37.0	98.00	54.98	*E. hirae* (confirmed)
*E. faecalis* OG1RF (GCF_000172575.2)	Complete	2.74	37.8	81.41	0.30	Different species
*E. faecium* DO (GCF_000173955.1)	Draft	2.29	49.0	70.33	0.00	Different species
*E. avium* ATCC 14025 (GCF_016726945.1)	Complete	2.55	43.7	72.19	0.01	Different species
*E. mundtii* ATCC 43186 (GCF_002845745.1)	Draft	6.20	62.3	70.39	0.00	Different species

ANI thresholds: ≥95% = same species, <95% = different species [20]. dDDH thresholds: ≥70% = same species, <70% = different species [38].

**Table 3:** Antimicrobial susceptibility results of *Enterococcus hirae* HI3.

Antibiotic Type	Antibiotics	Disk Content of Antimicrobials (μg/Disk)	Inhibition Zone Diameter (mm)	Strain Sensitivity
Beta-lactams	Penicillin (PEN)	10	27.17 ± 2.25	S
	Ampicillin (AMP)	10	28.17 ± 1.26	S
	Ceftriaxone (CTR)	30	16.17 ± 0.58	Not applicable
Macrolides	Erythromycin (E)	15	30.5 ± 0.87	S
Tetracycline	Tetracycline (TET)	30	6.0 (disk diameter)	R
Fluoroquinolones	Ciprofloxacin (CIP)	5	17.67 ± 0.58	I
Phenylpropanoids	Chloramphenicol (C)	30	31.17 ± 1.89	S
Sulfonamides	Co-trimoxazole (SXT)	25	6.0 (disk diameter)	R
Lincomycins	Lincomycin (MY)	2	6.0 (disk diameter)	R
Aminoglycosides	Gentamicin (GEN)	10	6.0 (disk diameter)	R

Note: R, resistant; S, susceptible; I, intermediate. Ceftriaxone may appear active *in vitro* against *Enterococcus* spp., but its clinical efficacy is not significant. Therefore, isolates should not be reported as susceptible to this agent (CLSI VET01S, 2024) [26]. The ceftriaxone inhibition zone diameter is provided for reference only. Accordingly, we have marked this result as “Not applicable” to avoid misinterpretation.

**Table 4:** Key genomic features of HI3.

Features	Genome Information
Total Length (bp)	3,055,857
N50 length (bp)	2,870,837
GC Content (%)	36.68
N rate (%)	0
Total genes	3061
Protein-coding genes	2972
tRNA	71
rRNA	18
CRISPR	1
Prophage	2
Antibiotic resistance gene	16
Virulence genes	20

**Table 5:** Probiotic-related genes predicted in the HI3 genome.

Predicted Function	KO ID	KO Name	KO Description	Number	Evidence for Probiotic Association
Bile salt stress resistance	K01442	*cbh*	Choloylglycine hydrolase	2	[39]
	K03530	*hupB*	HU family DNA-binding protein	1	[40]
Acid tolerance	K02759	*celC*	Cellobiose PTS system EIIA component	4	[27]
	K02760	*celA*	Cellobiose PTS system EIIB component	6	[27]
	K02761	*celB*	Cellobiose PTS system EIIC component	10	[27]
	K02108	*atpB*	F0F1 ATP synthase subunit A	1	[41]
	K02109	*atpF*	F0F1 ATP synthase subunit B	1	[41]
	K02110	*atpE*	F0F1 ATP synthase subunit C	1	[41]
	K02111	*atpA*	F0F1 ATP synthase subunit alpha	1	[41]
	K02112	*atpD*	F0F1 ATP synthase subunit beta	1	[41]
	K02113	*atpH*	F0F1 ATP synthase subunit delta	1	[41]
	K02114	*atpC*	F0F1 ATP synthase subunit epsilon	1	[41]
	K02115	*atpG*	F0F1 ATP synthase subunit gamma	1	[41]
	K03315	*nhaC*	Na^+^/H^+^ antiporter	2	[42]
Heat stress resistance	K03695	*clpB*	ATP-dependent Clp protease ATP-binding subunit ClpB	1	[43]
	K03544	*clpX*	ATP-dependent Clp protease ATP-binding subunit ClpX	1	[44]
	K01358	*clpP*	ATP-dependent Clp protease, protease subunit	2	[44]
	K04077	*groEL*	Chaperonin GroEL	1	[45]
	K04078	*groES*	Chaperonin GroES	1	[46]
	K03687	*grpE*	Molecular chaperone GrpE	1	[47]
	K04043	*dnaK*	Molecular chaperone DnaK	1	[43]
	K03686	*dnaJ*	Molecular chaperone DnaJ	1	[47]
	K03705	*hrcA*	Heat-inducible transcriptional repressor	1	[48]
	K03667	*hslU*	ATP-dependent protease HslVU ATPase subunit	1	[49]
	K01419	*hslV*	ATP-dependent protease HslVU peptidase subunit	1	[49]
Cold stress resistance	K03704	*cspA*	Cold shock protein	7	[50]
Surface adhesion	K19005	*ltaS*	Lipoteichoic acid synthase	2	[27]
	K01689	*eno*	Enolase 1/2/3	1	[51]
	K19420	*epsA*	tyrosine-protein kinase	4	[52]
	K00903	*epsB*	tyrosine-protein kinase	4	[52]
	K03217	*yidC*	YidC/Oxa1 family membrane protein insertase	2	[53]
	K07284	*srtA*	Surface protein transpeptidase	5	[54]
	K05896	*scpA*	Segregation and condensation protein A	1	[55]
	K06024	*scp* *B*	Segregation and condensation protein B	1	[55]
Oxidative stress resistance	K03741	*arsC*	Arsenate reductase	1	[29]
	K03671	*trxA*	Thioredoxin family protein	1	[56]
	K04564	*sod2*	Superoxide dismutase	1	[57]
	K00383	*GSR*	Glutathione–disulfide reductase	1	[58]
	K01919	*gshA*	Bifunctional glutamate–cysteine ligase GshA	1	[59]

**Table 6:** Putative antibiotic resistance genes identified in the genome of *E. hirae* HI3.

Gene ID	Gene	Predicted Antibiotics	Identity	Coverage	Position	Classification
SX000057	*Saur_fusA_FA*	fusidane antibiotic	74.89	99.85	Chromosome	Housekeeping
SX000058	*Efac_EFTu_GE2A*	elfamycin antibiotic	98.73	100	Chromosome	Housekeeping
SX000085	*Bsub_rpsE_SPT*	aminoglycoside antibiotic	80.72	100	Chromosome	Housekeeping
SX000203	*D-Ala-D-Ala ligase*	glycopeptide antibiotic	80.23	98.88	Chromosome	Housekeeping
SX000705	*eatAv*	pleuromutilin antibiotic	78.72	100	Chromosome	Housekeeping
SX000884	*Efac_liaF_DAP*	peptide antibiotic	87.65	100	Chromosome	Chromosomal efflux system
SX000885	*Efac_liaS_DAP*	peptide antibiotic	86.48	100	Chromosome	Chromosomal efflux system
SX000886	*Efac_liaR_DAP*	peptide antibiotic	95.71	99.05	Chromosome	Chromosomal efflux system
SX001011	*Efac_cls_DAP*	peptide antibiotic	86.54	100	Chromosome	Housekeeping
SX001026	*Efac_EFTu_GE2A*	elfamycin antibiotic	80.76	100	Chromosome	Housekeeping
SX002171	*efrA*	fluoroquinolone antibiotic; macrolide antibiotic; rifamycin antibiotic	83.63	100	Chromosome	Chromosomal efflux system
SX002280	*AAC(6′)-Iid*	aminoglycoside antibiotic	97.80	100	Chromosome	Intrinsic resistance
SX002700	*Saur_rpoC_DAP*	peptide antibiotic	72.80	97.68	Chromosome	Housekeeping
SX002701	*Bsub_rpoB_RIF*	peptide antibiotic; rifamycin antibiotic	76.20	96.68	Chromosome	Housekeeping
SX002950	*tet(M)*	tetracycline antibiotic	94.52	100	Plasmid 3	Acquired resistance gene
SX002952	*tet(L)*	tetracycline antibiotic	99.75	100	Plasmid 3	Acquired resistance gene

**Table 7:** Putative virulence factors in the *E. hirae* HI3 genome.

Gene ID	Gene	Predicted Functions	VFDB Category	Classification
SX000058	*tufA*	elongation factor Tu	Adherence	Moonlighting protein
SX000290	*bopD*	sugar-binding transcriptional regulator, LacI family	Biofilm	Transcriptional regulator
SX000526	*groEL*	chaperonin GroEL	Adherence	Moonlighting protein
SX000857	*rfbA*	glucose-1-phosphate thymidylyltransferase RfbA	ImmuneModulation	Exopolysaccharide biosynthesis
SX000859	*rfbB*	dTDP-glucose 4,6-dehydratase	ImmuneModulation	Exopolysaccharide biosynthesis
SX000973	*gndA*	NADP-dependent phosphogluconate dehydrogenase	ImmuneModulation	Metabolic enzyme
SX000974	*lisR*	two-component response regulator	Regulation	Stress response regulator
SX001026	*tufA*	elongation factor Tu	Adherence	Moonlighting protein
SX001698	*cpsB/cdsA*	phosphatidate cytidylyltransferase	ImmuneModulation	Exopolysaccharide biosynthesis
SX001699	*cpsA/uppS*	undecaprenyl diphosphate synthase	ImmuneModulation	Exopolysaccharide biosynthesis
SX001771	*bsh*	bile salt hydrolase	Bile salt hydrolase	Bile salt hydrolase, probiotic trait
SX001952	SGO_RS09900	polysaccharide biosynthesis protein	ImmuneModulation	Exopolysaccharide biosynthesis
SX001991	*galU*	UTP--glucose-1-phosphate uridylyltransferase GalU	ImmuneModulation	Exopolysaccharide biosynthesis
SX002047	*eno*	phosphopyruvate hydratase	Exoenzyme	Moonlighting protein
SX002050	*plr/gapA*	type I glyceraldehyde-3-phosphate dehydrogenase	Adherence	Moonlighting protein
SX002165	*clpP*	ATP-dependent Clp protease proteolytic subunit	StressSurvival	Stress response, protease
SX002231	*galU*	UTP--glucose-1-phosphate uridylyltransferase GalU	ImmuneModulation	Exopolysaccharide biosynthesis
SX002350	*efaA*	Endocarditis-specific antigen	Adherence	Cell wall adhesin
SX002594	EFMU0317_RS16950	C40 family peptidase	Adherence	Peptidase
SX002885	SPD_RS01770	nucleotide sugar dehydrogenase	ImmuneModulation	Exopolysaccharide biosynthesis

## Data Availability

The 16S rRNA gene sequence of *E.hirae* HI3 has been deposited in the NCBI database under the accession number PZ176951. The complete sequence of HI3 is available at GenBank under the accession numbers: JBWQFC010000001.1 (chromosome), JBWQFC010000002.1 (plasmid 1), JBWQFC010000003.1 (plasmid 2), and JBWQFC010000004.1 (plasmid 3).

## Supplementary Materials

The following supporting information can be downloaded at: https://www.mdpi.com/article/10.3390/microorganisms14081730/s1. Table S1: Comprehensive CARD annotation and four-category classification of all resistance-associated sequences identified in the *Enterococcus hirae* HI3 genome. Table S2: Annotation of coding sequences (CDSs) on plasmid 3 of *Enterococcus hirae* HI3. Table S3: NR database comparison of HI3 moonlighting proteins against pathogenic and non-pathogenic *Enterococcus* species.

## Abbreviations

The following abbreviations are used in this manuscript:

*Saur_fusA_FA*	*Staphylococcus aureus fusA* with a mutation conferring resistance to fusidic acid
*Efac_EFTu_GE2A*	*Enterococcus faecium* EF-Tu mutants conferring resistance to GE2270A
*Bsub_rpsE_SPT*	*Bacillus subtilis rpsE* mutations conferring resistance to spectinomycin
*Efac_liaF_DAP*	*Enterococcus faecium liaF* mutant conferring daptomycin resistance
*Efac_liaS_DAP*	*Enterococcus faecium liaS* mutant conferring daptomycin resistance
*Efac_liaR_DAP*	*Enterococcus faecium liaR* mutant conferring daptomycin resistance
*Efac_cls_DAP*	*Enterococcus faecium cls* conferring resistance to daptomycin
*Saur_rpoC_DAP*	*Staphylococcus aureus rpoC* conferring resistance to daptomycin
*Bsub_rpoB_RIF*	*Bacillus subtilis rpoB* mutants conferring resistance to rifampin
